# Pediatric Intranasal Lobular Capillary Hemangioma: A Rare Clinical Entity

**DOI:** 10.4274/balkanmedj.2017.0496

**Published:** 2017-12-01

**Authors:** Uğur Yıldırım, Rıfat Karlı, Seda Gün

**Affiliations:** 1 Department of Otolaryngology and Head and Neck Surgery, Ondokuz Mayıs University School of Medicine, Samsun, Turkey; 2 Department of Pathology, Ondokuz Mayıs University School of Medicine, Samsun, Turkey

A 9-year-old male presented to our clinic with symptoms of a right-sided intermittent epistaxis for the past one year. There was no headache, fever, trauma, nasal packing or history of foreign body. Besides there was no similar symptoms or illness in the family. On physical examination, both nasal cavities were normal in anterior rhinoscopy. The patient and her parents were informed about endoscopic nasal examination and after thier permittion, endoscopic nasal examination was performed. Endoscopic nasal examination revealed that a violescent mass which located between inferior turbinate and nasal septum at the posterior one-third of the right nasal cavity. The origin of the mass couldn’t be identified. The ear and throat examination was normal. Furthermore complete blood count and routine biochemical analysis were normal. Axial and coronal nasal and paranasal sinus computed tomography was performed. computed tomography revealed a right-sided soft tissue mass arising from posterior part of the inferior turbinate, and maxillary and ethmoid sinusitis (Figure 1).

Informed consent was taken from the patient’s parents. Endoscopic intranasal excision was performed under general anesthesia. In the right nasal cavity, there was a pedunculated, purplish, irregular necrotic mass which bleeds on touch originating from a region near the base at the posterior one-third of the nasal septum (Figure 1b). The lesion and the septal mucousa which was source of the mass was completely excised using cold dissection (Figure 1c). The patient was discharged without any complication two days after the operation. Histopathologic examination revealed lobular capillary hemangioma (Figure 1d). No recurrence was observed in the 3-month after the surgery.

Lobular capillary hemangioma which is also known as pyogenic granuloma typically occurs on the skin and in the oral cavity. Nasal cavity is an uncommon area for lobular capillary hemangioma (1). It is most frequently seen in the third and fifth decades of life, especially most commonly in women. Trauma and hormonal factors are considered for the etiology (2). Lobular capillary hemangioma is usually violescent, ulcerous, pedunculated or sessile lesions which bleeds on touch. It has variable sizes ranging from a few millimeters to a few centimeters. Nonspecific symptoms such as epistaxis, nasal obstruction, and purulant rhinorrhea have been reported in majority of the patients (3). Computed tomography or magnetic resonance imaging is useful for preoperative evaluation. Computed tomography is important for assessing of nasal and paranasal bone structures, particularly for the large-sized lesions which is originated from the nasal roof because the osseous destruction of the skull base can be seen (4). Endoscopic surgery is the prefered approach for the treatment (5).

## Figures and Tables

**Figure 1 a-e:**
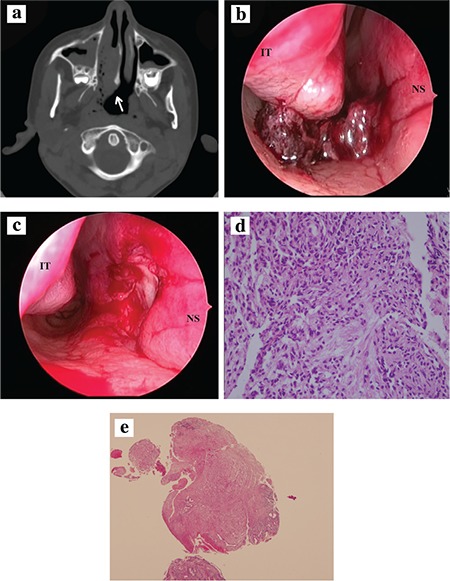
Computed tomography imaging shows a right-sided soft tisue mass and maxillary sinusitis (a), Hemorrhagic necrotic lesion originated from the posterior part of the nasal septum (b), Image shows the nasal septum after excision of the mass (c), A high-power view of a lobule shows compact proliferation of the capillaries (H&E x400) (d), Central vessel surrounded by lobules of endothelial-lined capillaries (H&E x40) (e). *NS: Nasal septum, IT: Inferior turbinate.*

## References

[1] Derkenne R, Coulet O, de Biasi C, Tomasi M (2012). Nasal cavity lobular capillary hemangioma due to insect sting. Eur Ann Otorhinolaryngol Head Neck Dis.

[2] Chi TH, Yuan CH, Chien ST (2014). Lobular capillary hemangioma of the nasal cavity: a retrospective study of 15 cases in taiwan.. Balkan Med J.

[3] Ozcan C, Apa DD, Görür K (2004). Pediatric lobular capillary hemangioma of the nasal cavity.. Eur Arch Otorhinolaryngol.

[4] Karagama YG, Howarth K, Steel PR, Spencer MG (2002). Lobular capillary haemangioma of the nasal vestibule: a rare entity.. Int J Pediatr Otorhinolaryngol.

[5] Hanazawa T, Yonekura S, Nakamura H, Fujikawa A, Okamoto Y (2016). Pre-operative effects of the administration of systemic corticosteroids combined with antibiotics on a lobular capillary hemangioma in the nasal cavity.. Auris Nasus Larynx.

